# A systematic review and meta-analysis of axillary intra-aortic balloon pump as a bridge to advanced heart failure therapy

**DOI:** 10.3389/fcvm.2026.1790627

**Published:** 2026-04-14

**Authors:** Haytham Allaham, Sara Kwiatkowski, Khalid Alswayed, Samuel Bennet, Olivia Cong, Laurence Magder, Gautam Ramani, Diljon Chahal, Mukta Srivastava, Seyed-Hossein Aalaei-Andabili, Cullen Soares, Anuj Gupta

**Affiliations:** 1 Division of Cardiovascular Medicine, Department of Medicine, University of Maryland Medical Center, Baltimore, MD, USA; 2 Division of Internal Medicine, Department of Medicine, University of Maryland Medical Center, Baltimore, MD, USA; 3 Department of Epidemiology & Public Health, University of Maryland Medical Center, Baltimore, MD, USA

**Keywords:** advanced heart failure, ambulation, axillary, IABP, intraaortic ballon pump, mechanical support, metanalisys

## Abstract

**Background:**

The axillary approach for intra-aortic balloon pump (IABP) placement offers an alternative to traditional transfemoral insertion, with the potential advantage of preserving mobility during temporary mechanical circulatory support.

**Methods:**

We performed a systematic review and meta-analysis of four retrospective studies evaluating outcomes in patients who received axillary IABP support.

**Results:**

Most patients supported with an axillary IABP were able to ambulate, with a pooled probability of 0.928 (95% CI: 0.811–0.999). The incidences of vascular complications (0.059; 95% CI: 0.001–0.137), stroke (0.022; 95% CI: 0.001–0.044), infection (0.037; 95% CI: 0.001–0.106), and bleeding (0.028; 95% CI: 0.005–0.052) were low. The most frequent adverse event was device failure (including kinking, rupture, migration, or malposition), with a pooled incidence of 0.314 (95% CI: 0.224–0.404).

**Conclusion:**

Axillary IABP support is a feasible bridge to advanced heart failure therapies, enabling ambulation in most patients and facilitating transition to definitive treatment. While the overall safety profile is favorable, the relatively high rate of device failure represents a key limitation of this approach.

## Introduction

The intra-aortic balloon pump (IABP), introduced in the 1960s, remains a widely utilized mechanical circulatory support modality for patients with advanced heart failure (HF) complicated by cardiogenic shock (CS), frequently serving as a bridge to transplantation or durable left ventricular assist device (LVAD) implantation (1–3). Notably, IABP support has been consistently associated with hemodynamic improvement, which correlates with reduced waitlist mortality and a lower incidence of decompensation among patients awaiting heart transplantation (4–6).

While femoral access remains the standard due to ease and safety, it restricts mobility and increases the risk of deconditioning during prolonged wait times. This can negatively affect outcomes, with physical activity and participation in physical therapy being key components of recovery in heart failure patients (7, 8). Axillary IABP placement, though technically more challenging, allows ambulation and may mitigate these risks. However, no randomized trials have compared femoral and axillary approaches, and their relative long-term outcomes remain uncertain. In this systematic review and meta-analysis, we examine outcomes and major complication rates reported in four key studies on axillary IABP, aiming to provide improved understanding of its role in this patient population.

## Materials and methods

This study is a systematic review and meta-analysis of four published studies evaluating outcomes and complication rates associated with axillary IABP placement in patients with HF. A systematic review of PubMed, Embase, Cochrane Central Register of Controlled Trials (CENTRAL), Web of Science (Clarivate Analytics), Science Direct, and Scopus (Elsevier) was performed on [March 11, 2025], for all studies performed between the year 1990–2025, using the search terms (“axillary intra-aortic balloon pump” OR “axillary IABP”) AND (“heart failure” OR “cardiogenic shock” OR “mechanical circulatory support”), with both MeSH terms and free-text keywords applied. Studies were selected according to the following inclusion criteria: (1) adult patients with HF supported with axillary IABP, (2) reported data on clinical outcomes and/or complication rates, and (3) publication in a peer-reviewed journal. Exclusion criteria included studies focusing solely on femoral IABP or lacking extractable outcome data.

A random-effects meta-analytic model was used to estimate pooled probabilities and 95% confidence intervals for key outcomes, accounting for inter-study variability. A random effects approach was utilized as we were not willing to assume that the probability of various outcomes would be the same in all four sites (an assumption made when using a fixed effects model). The random effects model will give somewhat more weight than a fixed effects model would to the smaller studies, as the random effects appropriately weigh the studies of different sizes based on the estimated heterogeneity. The model was fit using Proc GLIMMIX in SAS 9.4. The I2 statistics varied between outcomes but were generally quite low (<10%). As a result the random effects estimates converged to similar values that we would get using a fixed effects approach.

To evaluate for potential publication bias, we performed statistical assessments across all outcomes and complications included in the meta-analysis. For each study-specific endpoint, the event proportion was transformed using a logit transformation with Haldane–Anscombe continuity correction (adding 0.5 to events and 1 to total counts) to stabilize variances for proportions approaching 0% or 100%. Formal tests included Egger's linear regression test for funnel-plot asymmetry and Begg–Mazumdar rank-correlation test, applied to each outcome in all four studies. No evidence of small-study effects was identified (all Egger *p* ≥ 0.13; Begg *p* ≥ 0.33). However, because each analysis was based on a limited number of studies (k = 4), the statistical power of these tests remains inherently low, and absence of significance does not preclude subtle bias.

To further test robustness, we repeated the publication-bias assessment after excluding the smallest cohort (*Umakanthan* et al., *N* = 18). Using the same logit-transformed proportions with Haldane–Anscombe correction, Egger's and Begg's tests were recalculated for each outcome. The exclusion had minimal impact on statistical results (< 0.1 for all tests). Across all endpoints, the directionality remained unchanged, and no outcome demonstrated new evidence of asymmetry. The small absolute *p*-value shifts confirm that excluding the smallest study did not materially alter conclusions, supporting the stability of the findings.

This study was exempt from institutional review board approval, as it utilized publicly available data from previously published studies. We acknowledge the support of the University of Maryland, Baltimore, Institute for Clinical & Translational Research (ICTR) and the National Center for Advancing Translational Sciences (NCATS) Clinical Translational Science Award (CTSA), UM1TR004926.

## Results

**Figure 1:**
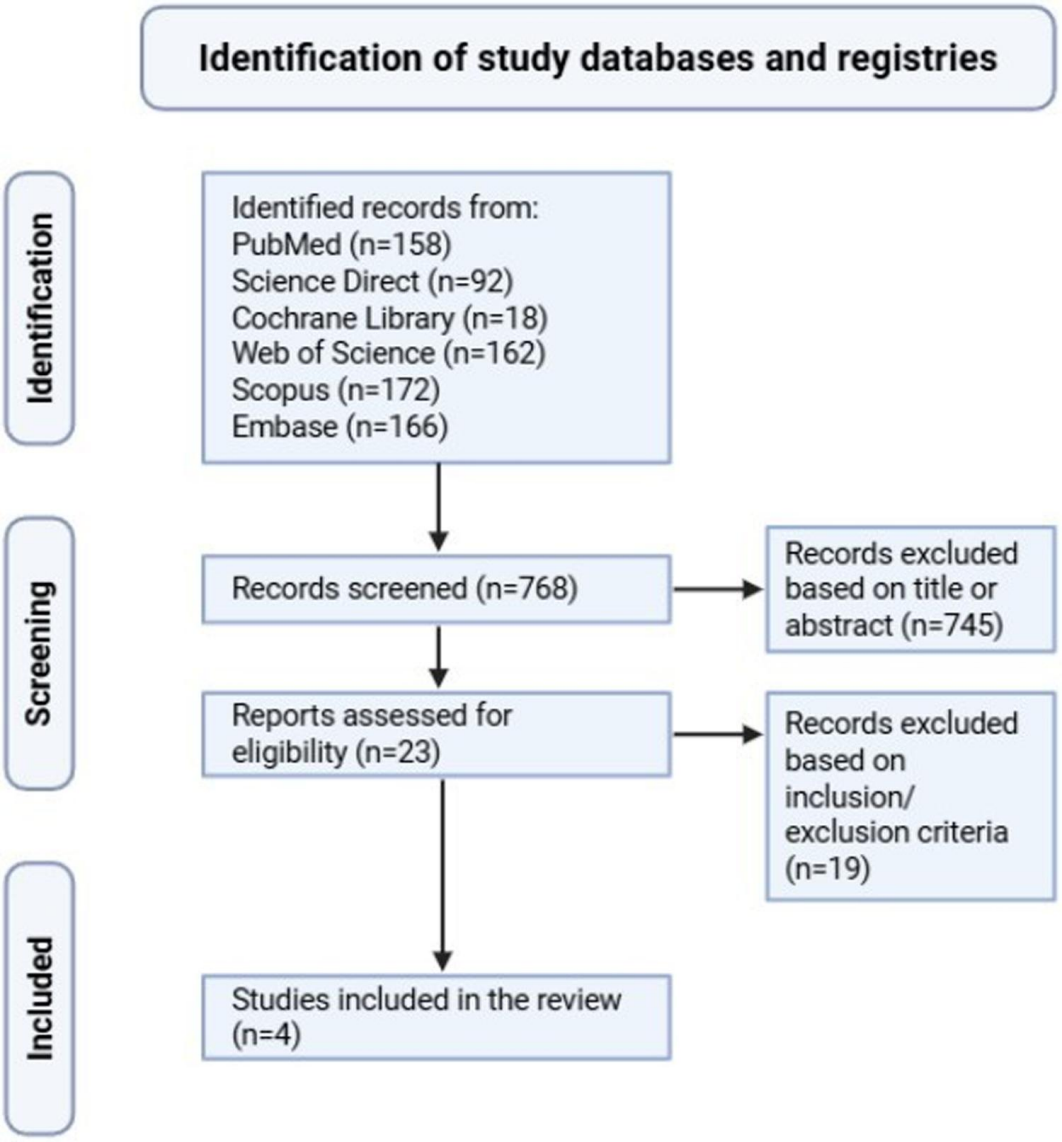
PRISMA flow diagram outlining the study selection process. A total of 768 records were identified through database searching. After removal of duplicates and initial screening of titles and abstracts, 28 articles were assessed for full-text eligibility. Of these, four retrospective observational studies met inclusion criteria and were included in the final analysis.

The initial literature search identified 768 records across six databases. Following title and abstract screening, 23 articles were selected for full-text eligibility assessment. Duplicate records and studies not meeting inclusion criteria were excluded. Ultimately, four retrospective studies were included, comprising a total of 492 patients (Figure 1). Key study characteristics are summarized in Table 1.

**Table 1:** Key characteristics of the studies used in the pooled analysis.

Author(s)	Published (Year)	Type of study	Sample size	Ischemic cardiomyopathy (%)	Median duration of IABP support (days)
Bhimaraj et al.	2020	Retrospective, single-center	195	58.5%	19
Nishida et al.	2022	Retrospective, single-center	241	43.2%	17
Umakanthan et al.	2012	Retrospective, single center	18	50%	19
Rosenbaum et al.	2021	Retrospective, multi-center	38	26.3%	7

### Outcomes of patients with axillary IABP

**Figure 2:**
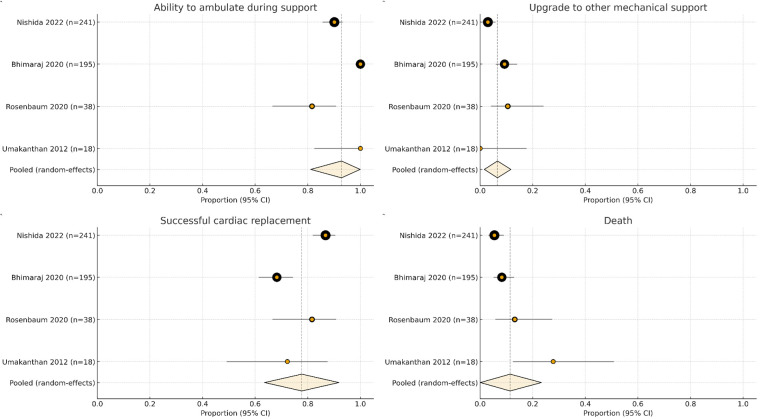
Forest plots summarizing outcomes associated with axillary IABP support across four observational studies, including ambulation capability, escalation to alternative mechanical circulatory support, successful bridging to heart transplantation, and death.

 Table 2 presents the pooled probabilities of key clinical outcomes in patients who received axillary IABP support, calculated using a random-effects model. Across all included studies, the ability to ambulate with an axillary IABP with a pooled probability of 0.928 (95% CI: 0.811–0.999). Similarly, a substantial proportion of patients were successfully bridged to transplantation, with a pooled probability of 0.777 (95% CI: 0.636–0.918). The incidence of death was low (0.114; 95% CI: 0.001–0.233), and only a small proportion of patients required escalation to LVAD or ECMO(0.066; 95% CI: 0.016–0.117) (Figure 2). In addition to pooled clinical outcomes, individual studies reported hemodynamic improvements following axillary IABP placement. As these measures were reported inconsistently across studies, they were not pooled but collectively support the observed hemodynamic benefits of axillary IABP.

**Table 2:** Estimate of the risk of various outcomes across four studies.

Event	Random Effects approach with 95% confidence interval.	Between-study variance (*τ*^2^)	Intra-class correlation (I^2^)
Ability to ambulate during support	0.928 (0.811, 0.999)	0.004	0.07
Upgrade to other mechanical support	0.066 (0.016, 0.117)	0.0004	0.01
Successful cardiac replacement	0.777 (0.636, 0.918)	0.005	0.03
Death	0.114 (0.001, 0.233)	0.003	0.04

### Risk of complications associated with axillary IABP

**Figure 3:**
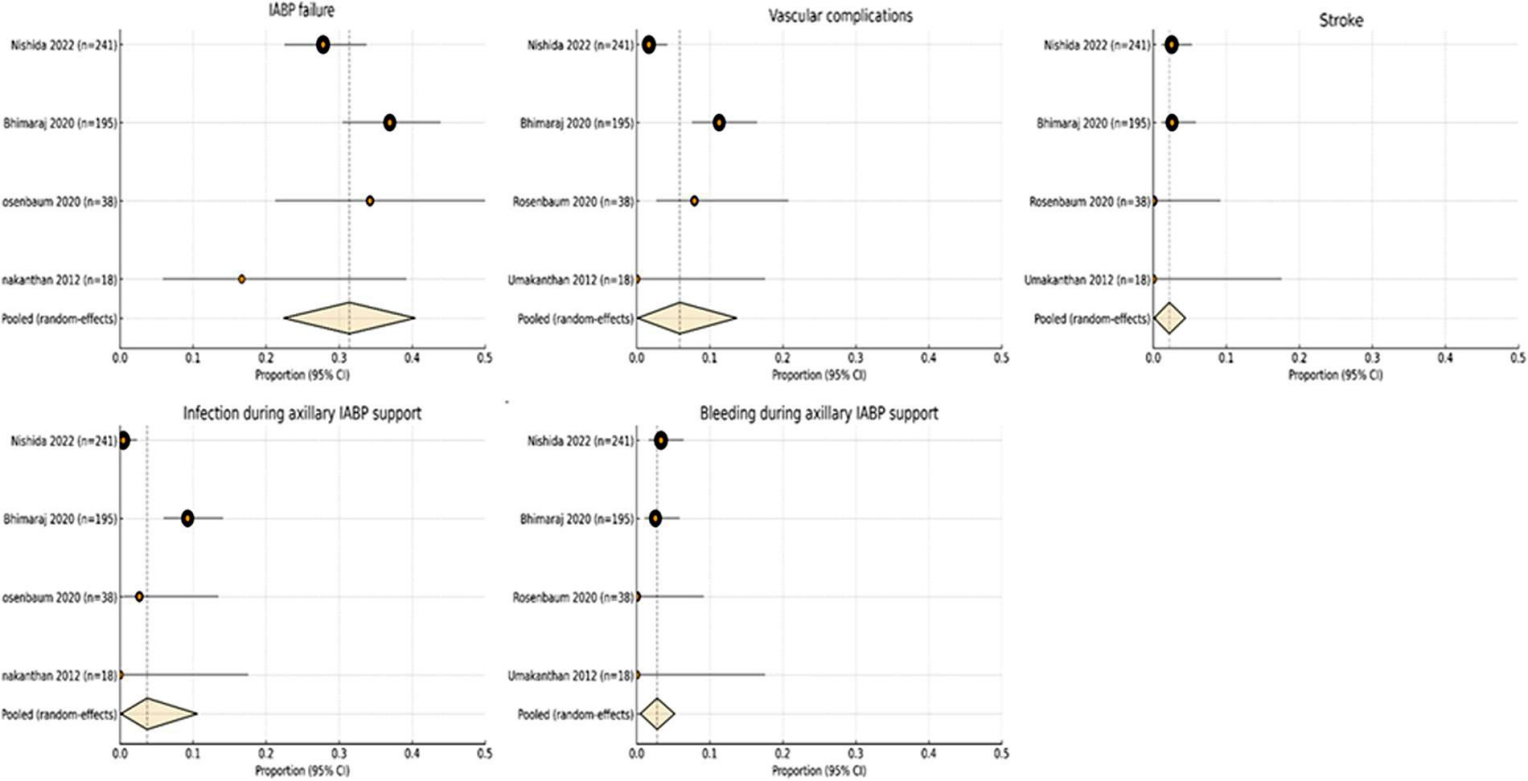
Forest plots illustrating the incidence of major complications across four observational studies and the pooled analysis of axillary IABP support.

 Table 3 summarizes the pooled complication rates associated with axillary IABP support. The rates of vascular complications (0.059; 95% CI: 0.001–0.137), stroke (0.022; 95% CI: 0.001–0.044), infection (0.037; 95% CI: 0.001–0.106), and bleeding (0.028; 95% CI: 0.005–0.052) were all low (Figure 3). The most notable complication was IABP device failure (kinking, rupture, migration, or malposition), with a pooled incidence of 0.314 (95% CI: 0.224–0.404), representing the most frequent adverse event across the included studies. While numerous studies have investigated vascular complications associated with femoral IABP support, data surrounding device failure rates in this patient population is scarce. Both Egger's regression test and Begg's rank correlation test (all *p* ≥ 0.13) demonstrated no evidence of small-study effects or publication bias across the evaluated clinical outcomes and complication endpoints associated with axillary IABP support (Table 4).

**Table 3:** Estimate of the risk of various complications across four studies.

Event	Random Effects approach with 95% confidence interval.	Between-study variance (τ^2^)	Intra-class correlation (I^2^)
IABP failure (kinking, migration, malposition, fracture, rupture)	0.314 (0.224, 0.404)	0.001	<0.01
Vascular Complications	0.059 (0.001, 0.137)	0.002	0.03
Stroke	0.022 (0.001, 0.044)	0.00	0.00
Infection	0.037 (0.001, 0.106)	0.001	0.03
Bleeding	0.028 (0.005, 0.052)	0.00	0.00

**Table 4:** Egger or begg test (all *p* ≥ 0.13), confirming no detectable small-study or publication bias.

Outcome	k (usable)	Egger p	Begg p
Ability to ambulate during support	4	0.415	0.750
Upgrade to other mechanical support	4	0.669	0.750
Successful cardiac replacement (transplant/LVAD/recovery)	4	0.848	1.000
Death	4	0.135	0.333
IABP failure (kinking, migration, malposition, fracture, rupture)	4	0.686	0.750
Vascular complication	4	0.397	0.750
Stroke	4	0.288	0.750
Bacteremia/infection	4	0.204	0.750
Bleeding	4	0.225	0.333

## Discussion

The most common method for percutaneous IABP placement is through the femoral artery. However, a significant drawback to this approach is its limitation on patient mobility, which can lead to deconditioning. In recent years, the number of patients listed as Status 2 has increased, resulting in longer waitlist times and, consequently, extended periods of patients remaining bedridden while supported by a transfemoral IAB P (9). It has been reported that just 5 days of muscle disuse can result in notable loss of skeletal muscle mass and strength (10). Given this, the American College of Cardiology/American Heart Association recommends physical therapy as a critical component of heart failure management (8). Encouraging early ambulation is essential, as physical therapy offers benefits such as improved endothelial function, reduced intrinsic catecholamine levels, and enhanced oxygen uptake – factors that can have a synergistic impact on the outcomes of critically ill heart failure patients (11, 12).

To address the mobility limitations of the transfemoral approach, McBride and colleagues introduced the axillary approach in 1989 (13). This method's key advantage is its ability to enable patient mobilization, a feature that sets it apart from the femoral approach. In a retrospective analysis by Umakanthan et al., all 18 patients with axillary IABP maintained their ability to ambulate, with walking distance improving significantly from 400 feet to 2700 feet (14). Similarly, Nishida and colleagues found that 217 out of 241 patients with axillary IABP were able to ambulate and showed an improvement in walking distance after the procedure (15).

Our study further supports these findings, showing that a significant proportion of patients (92.8%; CI 0.811–0.999) with axillary IABP maintain their mobility. Of note, the definition and reporting of ambulation varied across studies. Nishida et al. reported that 90% of patients were able to walk with assistance, with a significant increase in walking distance over the study period (15). Similarly, Umakanthan et al. described an aggressive ambulation protocol initiated early after device placement, with serial assessment demonstrating substantial increases in daily walking distance (14). In contrast, Bhimaraj et al. reported ambulation within structured physical therapy protocols, with patients typically mobilized out of bed within 24 h and ambulating within 48 h, without standardized quantification of distance (16). Rosenbaum et al. likewise defined ambulation more broadly as the ability to be mobile during support, reporting that the majority of patients were ambulatory without providing specific distance metrics (17). Collectively, these findings indicate that while ambulation is consistently feasible with axillary IABP support, the outcome reflects heterogeneous definitions ranging from assisted mobility to quantitatively measured walking distance.

### Complications of axillary IABP

While axillary IABP therapy enables excellent mobility, it is associated with a unique set of complications.

Device failure is the major drawback of axillary IABP use in clinical practice. Common causes include kinking, migration, and malposition of the device, which often necessitate fluoroscopic repositioning or device removal. The relatively high incidence of device failure may be attributable to the angulated and tortuous course of the axillary and left subclavian arteries as they originate from the aortic arch. Additionally, excessive upper extremity movement can further predispose to displacement and migration of the device. This is consistent with the current meta-analysis findings, as the incidence of device failure was noted to be 0.314 (95% CI: 0.224–0.404) across the studies included.

Across the included studies in the meta-analysis, the reported IABP failure rate represents a heterogeneous composite of device-related events with differing clinical severity. In Nishida et al., failure was primarily driven by mechanical complications, including balloon displacement (8.7%), kinking (7.5%), and rupture (5.0%), all of which required device exchange but were generally manageable without termination of support (15). Similarly, Rosenbaum et al. reported that 21.4% of devices experienced balloon failure or migration necessitating replacement, though no major adverse clinical sequelae were observed (17). In contrast, Umakanthan et al. reported minimal major device-related complications despite prolonged ambulatory support, reflecting a lower burden of mechanical failure (14). Across studies, these events were typically addressed with repositioning or device exchange and rarely resulted in discontinuation of therapy. Collectively, these findings indicate that the composite “failure” endpoint is largely driven by manageable mechanical issues rather than clinically significant failure to achieve therapeutic goals.

Earlier studies have suggested an increased risk of stroke with axillary IABP use (15). Subsequent analyses indicate that this risk is primarily associated with right-sided axillary insertion, likely due to the shared origin of the right subclavian, right common carotid, and brachiocephalic arteries, which facilitates cerebral embolization. In addition, advancing the catheter through the aortic arch during right-sided placement may compromise flow to the left common carotid artery. Among the included studies in this meta-analysis, reporting of axillary access laterality was variable. Nishida et al. reported both right- and left-sided insertions (61% vs 39%, respectively), reflecting earlier institutional practice (15). In contrast, other series demonstrated a clear preference for left-sided access, with both Umakanthan et al. and Rosenbaum et al. reporting exclusive use of the left axillary artery (14, 17). Bhimaraj et al. did not specify access laterality (16). Collectively, these findings suggest a temporal shift toward left-sided insertion, likely driven by anatomical considerations and potential reduction in cerebrovascular risk.

An additional limitation of the axillary approach is that device removal may necessitate general anesthesia, particularly in cases requiring surgical cutdown or vascular repair. This contrasts with femoral access, where removal is more commonly performed at the bedside. Although less invasive removal strategies may be feasible in select axillary cases depending on institutional expertise, this consideration may impact procedural planning, resource utilization, and patient recovery.

The current meta-analysis demonstrated a low incidence of bleeding, hematoma, infection, and vascular complications associated with axillary IABP. These findings are consistent with clinical practice and support a safety profile comparable to that of the traditional femoral approach. However, further studies directly comparing axillary and femoral access are warranted to more precisely define the relative risk of complications for each approach.

### Ambulatory femoral IABPs as an alternative

Traditional femoral IABP restricts patient mobility, whereas axillary access enables ambulation but may be associated with higher device-related complications, as observed in this analysis. An alternative approach is the use of structured ambulatory femoral IABP protocols, which incorporate staged mobilization and multidisciplinary oversight and have demonstrated feasibility and acceptable safety profiles in selected patient populations, although the supporting evidence remains limited and largely observational (18–21).

Successful implementation of an ambulatory femoral IABP program requires a coordinated multidisciplinary approach, including experienced physical therapy support and vigilant critical care nursing. Structured protocols are essential. For example, Skrzat et al. described a phased mobilization strategy incorporating a standardized mobility checklist, demonstrating that approximately 50% of patients with femoral IABP were able to ambulate without major complications (22). Ongoing nursing education and the use of standardized protocols for early recognition of device-related issues may further reduce complication risk (23).

Beyond IABP, other mechanical circulatory support strategies, including transvalvular microaxial pumps (e.g., Impella) and venoarterial extracorporeal membrane oxygenation (VA-ECMO), may be considered based on the severity of hemodynamic compromise, need for left ventricular unloading, and overall clinical trajectory (24, 25). However, these modalities carry distinct risk profiles, including higher rates of vascular and bleeding complications, and are typically reserved for more advanced shock states. Overall, selection of support strategy should be individualized, and adequately powered comparative studies are needed to define the optimal approach for ambulatory support in advanced heart failure.

## Conclusion

This systematic review and meta-analysis demonstrate that axillary intra-aortic balloon pump support is a feasible bridge to advanced heart failure therapies, with most patients able to ambulate during support and successfully transition to definitive treatment. Despite its favorable safety profile, device failure remains an important limitation of the axillary approach, which is critical limitation in their widespread use in clinical practice. Structured femoral ambulation protocols may offer a promising alternative by combining the benefits of mobility with potentially lower device failure risk. Further studies are needed to define the safety, feasibility, and comparative outcomes of ambulatory femoral vs. axillary IABP strategies.

### Study limitations

This study has several important limitations. Foremost, it is based on a systematic review and meta-analysis of only four small retrospective studies, without any randomized clinical trial data. The inherent selection bias and lack of standardized protocols across these studies substantially weaken the strength and reliability of the pooled conclusions. Therefore, larger, prospective, and ideally randomized studies are essential to establish more definitive evidence. Second, combining data from different study designs and populations can introduce heterogeneity, potentially reducing the reliability of the overall findings. Additionally, there was insufficient comparative data from patients supported with femoral IABPs, limiting the ability to directly evaluate outcome differences between axillary and femoral approaches. In terms of outcomes, variables such as device failure were not consistently defined across studies, which may limit the comparability and precision of pooled estimates. Finally, the definition of ambulation was not consistent between the studies. As such, these limitations warrant cautious interpretation of the current meta-analysis findings.

## Data Availability

The original contributions presented in the study are included in the article/Supplementary Material, further inquiries can be directed to the corresponding author.
